# Uniform trichromacy in *Alouatta caraya* and *Alouatta seniculus*: behavioural and genetic colour vision evaluation

**DOI:** 10.1186/s12983-021-00421-0

**Published:** 2021-07-08

**Authors:** Leonardo Dutra Henriques, Einat Hauzman, Daniela Maria Oliveira Bonci, Belinda S. W. Chang, José Augusto Pereira Carneiro Muniz, Givago da Silva Souza, Luiz Carlos de Lima Silveira, Olavo de Faria Galvão, Paulo Roney Kilpp Goulart, Dora Fix Ventura

**Affiliations:** 1 Departamento de Psicologia Experimental, Instituto de Psicologia, Universidade de São Paulo, São Paulo, Brazil; 2 Instituto de Ensino e Pesquisa, Hospital Israelita Albert Einstein, São Paulo, Brazil; 3 Department of Cell and System Biology, University of Toronto, Toronto, Canada; 4 Centro Nacional de Primatas, Instituto Evandro Chagas, Ministério da Saúde, Ananindeua, Pará Brazil; 5 Núcleo de Medicina Tropical, Universidade Federal do Pará, Belém, Pará Brazil; 6 Instituto de Ciências Biológicas, Universidade Federal do Pará, Belém, Pará Brazil; 7 Núcleo de Teoria e Pesquisa do Comportamento, Universidade Federal do Pará, Belém, Pará Brazil

**Keywords:** Uniform Trichromacy, Platyrrhini, Colour discrimination ellipses, Positive reinforcement, Cambridge colour test, Neotropical primates

## Abstract

Primate colour vision depends on a matrix of photoreceptors, a neuronal post receptoral structure and a combination of genes that culminate in different sensitivity through the visual spectrum. Along with a common cone opsin gene for short wavelengths (*sws1*), Neotropical primates (Platyrrhini) have only one cone opsin gene for medium-long wavelengths (*mws/lws*) per X chromosome while Paleotropical primates (Catarrhini), including humans, have two active genes. Therefore, while female platyrrhines may be trichromats, males are always dichromats. The genus *Alouatta* is inferred to be an exception to this rule, as electrophysiological, behavioural and molecular analyses indicated a potential for male trichromacy in this genus. However, it is very important to ascertain by a combination of genetic and behavioural analyses whether this potential translates in terms of colour discrimination capability. We evaluated two howler monkeys (*Alouatta* spp.), one male *A. caraya* and one female *A. seniculus*, using a combination of genetic analysis of the opsin gene sequences and a behavioral colour discrimination test not previously used in this genus. Both individuals completed the behavioural test with performances typical of trichromatic colour vision and the genetic analysis of the *sws1*, *mws*, and *lws* opsin genes revealed three different opsin sequences in both subjects. These results are consistent with uniform trichromacy in both male and female, with presumed spectral sensitivity peaks similar to Catarrhini, at ~ 430 nm, 532 nm, and 563 nm for S-, M- and L-cones, respectively.

**Supplementary Information:**

The online version contains supplementary material available at 10.1186/s12983-021-00421-0.

## Background

Colour vision is thought to play an important role in the survival of primates. The ability to visually discriminate a target from the background by hue differences may improve the capacity to find food sources and to detect predators [1, 2]. Most mammals have dichromatic colour vision, with two types of cones that contain different opsins that absorb photons at distinct wavelengths. In mammals (eutherians and marsupials), a long/middle wavelength sensitive opsin (LWS/MWS) absorbs photons at the red/green region of the visible light spectrum, and a short-wavelength sensitive opsin (SWS1) has absorption peaks (λ_max_) that vary from the UV to the violet region of the spectrum [3].

In the lineage leading to the Paleotropical primates (Parvorder Catarrhini), which includes humans, a duplication event of the long-wavelength sensitive opsin gene, *lws*, located in the X chromosome, followed by subsequent mutations, led to the rise of two distinct opsins maximally sensitive to middle and to long wavelengths (OPN1MW, MWS and OPN1LW, LWS). Along with the SWS1 opsin, the presence of MWS and LWS opsins enables uniform trichromatic colour vision among males and females of Catarrhini. On the other hand, Neotropical primates (Parvorder Platyrrhini), like most other placental mammals, have one copy of the *lws* opsin gene in the X chromosome, meaning that both, males, with a single X chromosome, and homozygous females, are dichromats. However, allelic polymorphisms of the LWS is observed in many species of Platyrrhini, leading to a variety of colour vision phenotypes among different species and among individuals of a same species [4]. These polymorphisms may lead to trichromacy in heterozygous females, with two distinct *lws* opsin genes in each X chromosome that are translated into opsins that can be spectrally tuned toward the green (middle wavelengths) or the red (long wavelengths) regions of the light spectrum [5].

Howler monkeys (genus *Alouatta*) are unique among the Platyrrhini and even within the Atelidae Family. They display a distinct architecture of the skull and mandible that have evolved in association with behavioural changes, such as an increased folivorous diet, vocal communication and energy-minimizing strategies [6]. Jacobs and collaborators [7] found the first evidence of uniform colour vision among males and females of *Alouatta* spp., using electroretinogram (ERG) and molecular analysis of the opsin genes of two males and one female of red howler monkey (*A. seniculus*) and four males and one female of black howler monkey (*A. caraya*). They found evidence of two distinct opsins, sensitive to middle and long wavelengths, MWS and LWS, with absorbance peaks at 530 and 562 nm (λ_max_), respectively, in males and females of both species. Based on these intriguing findings, several studies have been carried out on howler monkeys’ vision. Some differences between howler monkeys and other Platyrrhini include a higher peak density of cones in the fovea and lower cone density in the retina periphery, however, with no change in the overall total photoreceptor count or in the density of bipolar and ganglion cells [8–12]. These features indicate a photoreceptor compaction at the central retina, and its effect on colour vision is unclear. Silveira and collaborators [12] have performed microspectrophotometry (MSP) analysis in the retinas of two males of black howler monkeys (*A. caraya*) and found spectral absorption peaks at 530 and 558 nm, similar to those found with ERG by Jacobs and collaborators [7].

Matsushita and collaborators [13] investigated the *lws* and *mws* opsin genes of the mantled howler monkey (*A. palliata*) and the Guatemalan black howler monkey (*A. pigra*), native from Central America. The authors found evidence of trichromacy in males and females, based on genetic analysis. They reported the presence of OPN1MW/ OPN1LW hybrid genes with estimated absorption peaks at 547 nm for *A. palliata* hybrid and 546 nm for *A. pigra* hybrid [14]. Araújo and collaborators [15] performed the first behavioural investigation of the predicted potential for trichromacy. In this study, four *A. caraya*, three males and one female, performed a discrimination task involving 16 pairs of Munsell colour chips, in which selected chromaticities were shown over a black background. The authors found that males of *A. caraya* performed similarly to trichromatic females of other platyrrhine species, also indicating uniform colour vision between males and females of *A. caraya*.

In their behavioural study, Araújo Jr. and collaborators [15] used the Munsell colour chips with random brightness values to avoid discrimination being based on brightness cues rather than colour [16, 17]. However, pseudoisochromatic stimuli offer a more robust testing paradigm for controlling other visual cues, by using spatial and luminance noise to avoid cues that could allow non-uniform trichromats to discriminate a target from the background based on non-chromatic differences. Pseudoisochromatic tests have already been used to test colour vision in other genera of Platyrrhini, including squirrel monkeys (*Saimiri* sp.) and capuchin monkeys (*Cebus* sp. and *Sapajus* spp) [18–20], as well as an albino *Sapajus apella* subject [21].

In order to further understand *Alouatta* colour vision capabilities, we evaluated the colour vision of one male black howler monkey (*A. caraya*) and one female red howler monkey (*A. seniculus*), with a computerized pseudoisochromactic colour vision test, applied for the first time in howler monkeys. This test allows the quantitative identification of colour discrimination thresholds. We also performed molecular analysis of the opsin genes using cloning techniques and polymerase chain reactions (PCR), which allowed us to identify the amino acid residues responsible for the opsins spectral tuning, and to infer their absorbance peaks. This is the first study to combine a robust behavioural approach with molecular techniques to investigate the molecular basis of the colour vision phenotype in this unique group of Platyrrhini.

## Results

### Procedure

The behavioural experiments were limited by the difficulty in attracting the *Alouatta* monkeys to engage and perform the tests. This was remarkably different from the behaviour of other Platyrrhini, such as *Sapajus* investigated previously by our group [20, 21]. Some alternative training techniques and rewards such as grapes, nuts, 190 mg and 300 mg banana pellets were tested, until we reached the training protocol described in the Methods section. From the six subjects initially trained, only three reached the test phase, and only two finished all vectors from the three chromatic discrimination ellipses, after 26 months of training.

### Genetic analysis

For three subjects that reached the behavioural colour discrimination tests phase, two *A. caraya* males and one *A. seniculus* female, the exons 3 and 5 of the *lws/mws* opsin genes were sequenced. The sequences of those subjects were the same at the critical amino acid residues responsible for the spectral shifts between the LWS and MWS opsins [22].

**Fig. 1:**
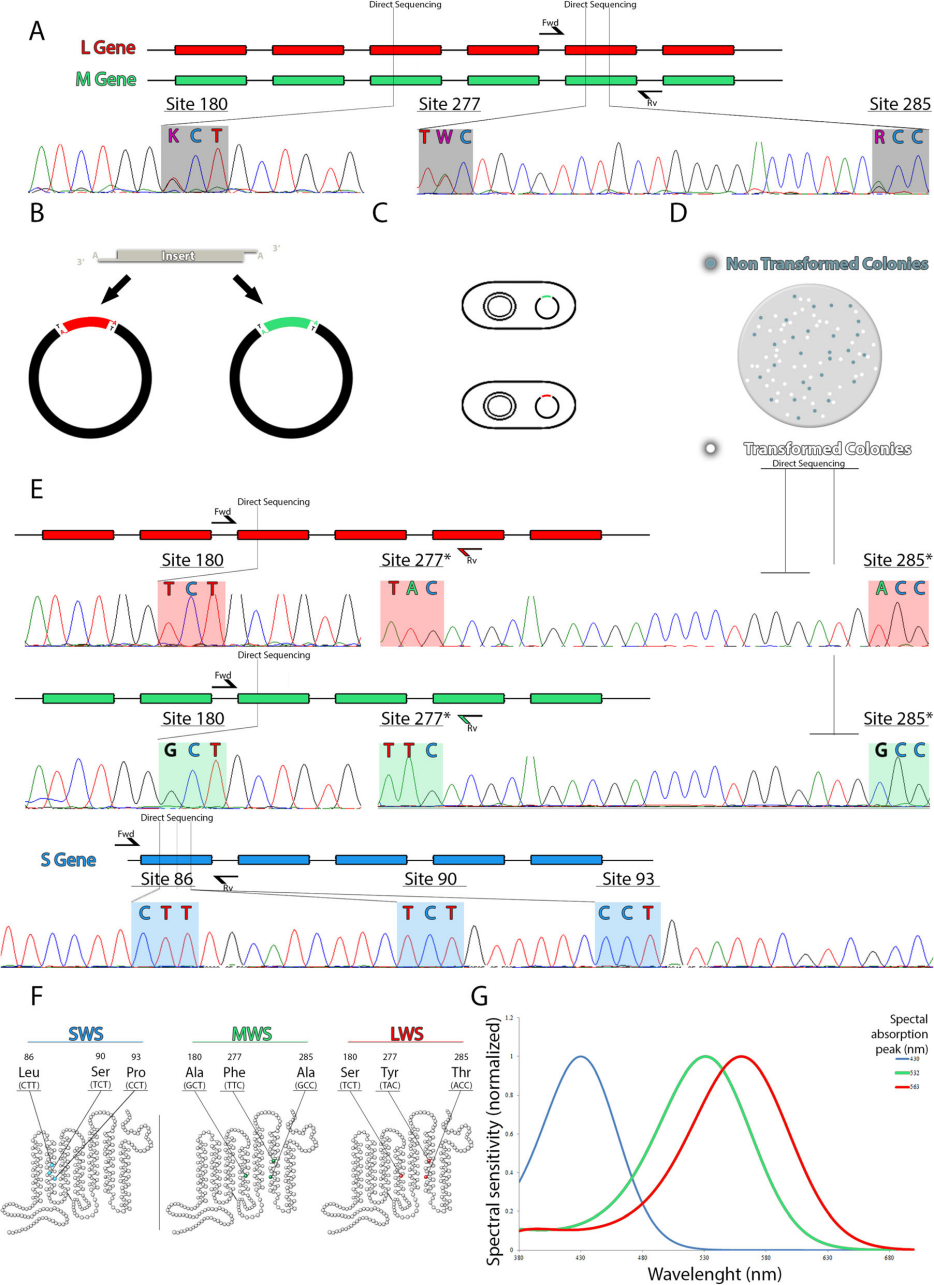
Molecular biology procedures used to estimate the spectral sensitivity curves of the three opsins of *Alouatta caraya* and *Alouatta seniculus*. This figure is a representation - proportion and scales are oversimplifications for better readability. **A** Exons 3 and 5 of the *mws* and *lws* opsin genes were amplified by PCR, directly sequenced, and the resulting chromatograms showed heterozygous sites (highlighted in grey). **B** The amplified exon 5 of the *mws* and *lws* genes were inserted into plasmid vectors, which were used to transform competent *E. coli* (**C**). **D** Bacterial colonies were white-blue screened, and multiple individual white clones were grown, purified and sequenced. **E** Sequencing results enabled us to identify the exon 5 of each gene, separately, and to design reverse primers specific for each gene in the exon 5. Exons 3 to 5 of *mws* and *lws* genes were amplified using the forward primers for exon 3 in each gene and the reverse primers designed in the exon 5. Exon 1 of *sws1* gene were sequenced in order to identify the amino acids at the critical spectral tuning sites. *The chromatogram of the exon 5 of *mws* and *lws* were drawn from reverse sequences. In order to keep the nomenclature, standard the codon sequences were presented. **F**, **G** Based on the amino acids located at the spectral tuning sites we predicted the spectral sensitivity peak of each opsin. **G** Spectral absorbance curves were inferred from the spectral peaks and based on Stockman and Sharpe [23] template

Sequencing analysis from 10 individual clones of each subject obtained from the amplified exon 5 indicated the presence of one opsin with residues F277 and A285, and one opsin with the amino acid combination Y277 and T285. In order to determine the combination of amino acids at the three spectral tuning sites, 180, 277, and 285, we performed PCRs encompassing exons 3 to 5 with specific reverse primers for each opsin gene. We obtained a PCR product over 2 kb, and sequencing results enabled us to identify the beginning (exon 3) and the end (exon 5) of each sequence, and to determine the correct combination of amino acids of each opsin. The MWS opsin had the amino acids A180, F277, and A285, which allowed us to estimate a λ_max_ at 530 nm. The LWS opsin, with residues S180, Y277, and T285, had an estimated λ_max_ at 563 nm (Fig. 1). Therefore, our results from genetic analysis confirmed the presence of two distinct opsin genes, *mws* and *lws*, in both males and females individuals of the *Alouatta* genus*.*

The sequence of the *sws1* gene of the three subjects was also identified and found to be identical in males and females. The amino acids at the critical positions for spectral tuning showed the same sequence as *Saimiri boliviensis* and *Callithrix jacchus* (Fig. 1 E). For the SWS1 opsin, the spectral absorption peak was inferred based on residues I46, L49, L52 V81, L86, S90, P93, G114, L116, and T118 (Fig. 1 E). Based on previous studies, these residues allowed us to estimate a spectral absorption peak between 423 and 430 nm (Fig. 1 G).

### Behavioural tests

**Fig. 2:**
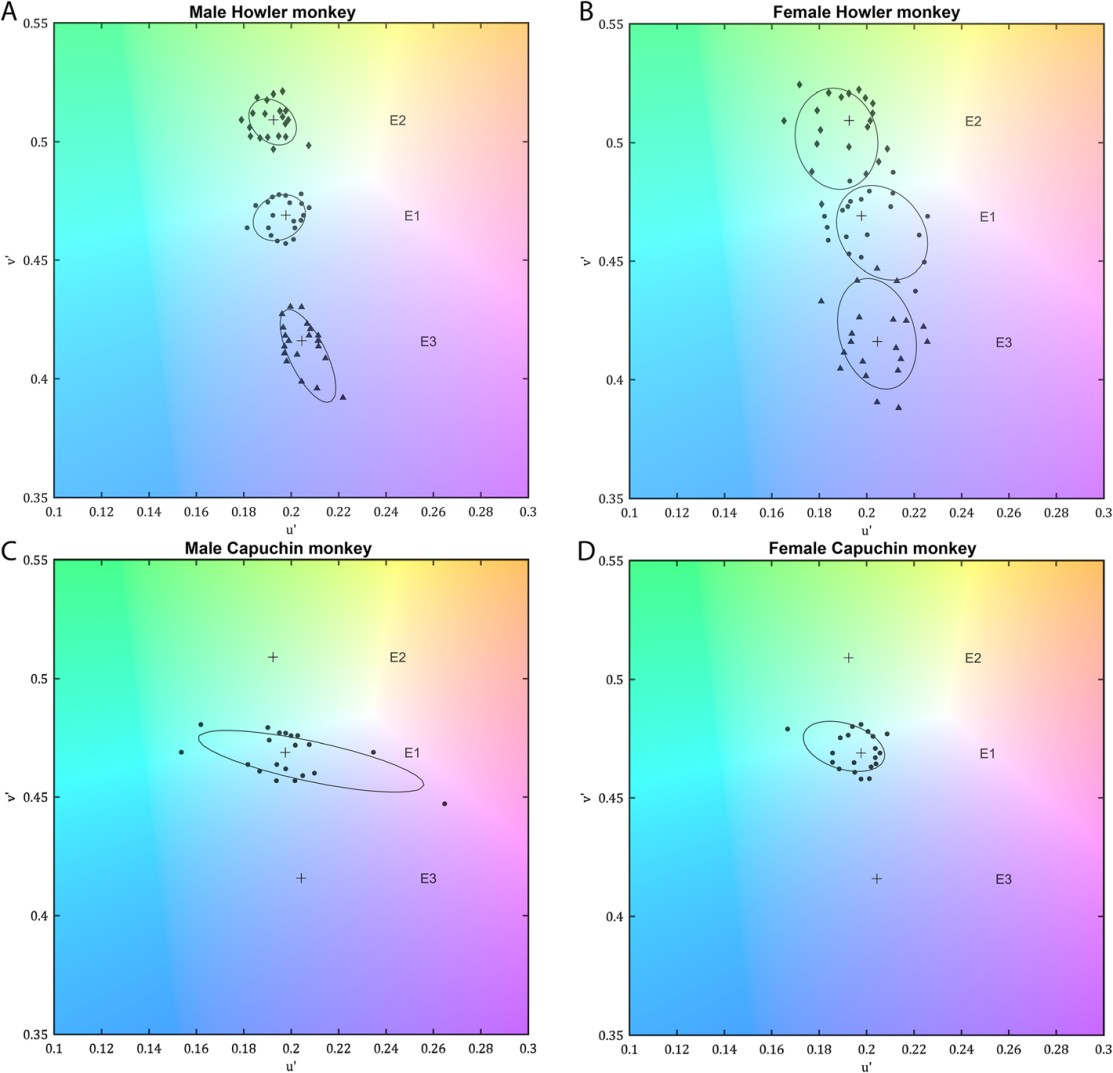
Colour vision evaluation. Colour discrimination ellipses estimated from (**A**) a trichromatic male of black howler monkey (*Alouatta caraya*), (**B**) a trichromatic female of red howler monkey (*A. seniculus*), (**C**) a dichromatic male capuchin monkey (*Sapajus* sp.) and (**D**) a trichromatic female capuchin monkey (*Sapajus* sp.). The capuchin monkeys were evaluated in previous work from our group [21, 25], were tested using the same apparatus for only one of the background chromaticities, and were included to allow comparison with the Alouatta phenotype. Black circles show the fitted ellipses for the group of chromatic vector thresholds tested at each background chromaticity. Thresholds correspond to vector lengths in u’v’ units of the 1976 CIE chromaticity diagram along each of 20 equally spaced vectors connecting to the background chromaticity. The crosses represent the background chromaticity for each ellipse, and the tested vectors thresholds for the first background (E1, u’ = 0.1977, v’ = 0.4689) are shown with black dots, for the second (E2, u’ = 0.1925, v’ = 0.5092) with black diamonds and for the third (E3, u’ = 0.2044, v’ = 0.4160) with black triangles

Colour discrimination thresholds for the 20 chromatic vectors (Table 1) were fitted to ellipse functions whose parameters allow diagnosing colour vision deficiencies. In general terms, best-fit ellipses characteristic of dichromats have a markedly elongated shape, due to the occurrence of test vectors with considerably higher thresholds compared to the other vectors. In turn, the typical ellipses of trichromats have an approximately circular shape, due to uniformly low thresholds in all test vectors. Ellipse parameters are described in Table 2. The confusion axis angle, major axis length and axis ratio of the *Alouatta* subjects were similar to that of trichromatic subjects. When compared to the ellipsis fit of colour discrimination thresholds of capuchin subjects tested using the same protocol (Fig. 2), the male capuchin monkey ellipsis (2 C) is elongated with higher thresholds in some directions, whereas male and female howler monkey and the trichomatic female capuchin monkey display a more round ellipsis fit with tresholds similar in all directions, indicating that both individuals have functional MWS, LWS, and SWS opsins.

**Table 1:** Colour discrimination thresholds summary from the male *Alouatta caraya* and female *Alouatta seniculus*

Subject	Mean (u’v’ unit)	Standard deviation
Male *Alouatta caraya*		
Ellipse 1	0.010475	0.004822
Ellipse 2	0.00921	0.003624
Ellipse 3	0.010955	0.006259
Female *Alouatta seniculus*		
Ellipse 1	0.016775	0.008880
Ellipse 2	0.016935	0.007730
Ellipse 3	0.01839	0.007384

Subjects were tested using an adaptation from Goulart et al. [24] colour vision test, for the *Alouatta* genus. Mean vector length threshold of the 20 vectors for each center displayed in CIE 1976 u’v’ CIE chromaticity diagram units

**Table 2:** Parameters of colour discrimination ellipses fits for a male *Alouatta caraya* and a female *Alouatta seniculus*

Subject	Length (u’v’ unit)	Ratio	Angle (degree)
Male *Alouatta caraya*			
Ellipse 1	0.022158	1.2608	28.053
Ellipse 2	0.022844	1.3323	138.134
Ellipse 3	0.040704	2.7798	116.07
Female *Alouatta seniculus*			
Ellipse 1	0.043602	1.2659	130.95
Ellipse 2	0.04284	1.241	101.06
Ellipse 3	0.048058	1.5154	106.87

Subjects were tested using an adaptation from Goulart et al. [24] colour vision test, for the *Alouatta* genus. Length = Major axis of the ellipses in u’v’units of CIE 1976 chromaticity diagram units, Ratio = major axis length over minor axis length, Angle = relative angle of the major axis in degrees

## Discussion

Our results strongly support the hypothesis of uniform trichromacy in *A. caraya* and *A. seniculus*. The genetic analysis showed the presence of *mws*, *lws*, and *sws1* opsin genes in the genome, and confirmed the presence of two different opsins sensitive to long and to middle wavelengths, in both male and female, similar to the findings of Jacobs and collaborators [7]. We found no signals of hybrid *mws*/*lws* gene as reported by Matsushita and collaborators [13] for the species from Central America, *A. palliata* and *A. pigra*. However, our sample is too small to address about the presence of hybrid genes of approximately 13% [14], so further populational studies should be performed to evaluate this hypothesis. The spectral absorbance peaks inferred in our study for the M (530 nm) and L (563 nm) opsins of *A. caraya* and *A. seniculus*, were similar to the values obtained from ERG (530 and 562 nm) [7] and MSP (530 and 558 nm) [12]. Our molecular approach, i.e. cloning the exon 5 of the genes into plasmid vectors and further use of specific primers for PCRs from exon 3 to 5, was successful in achieving the subjects’ sequence and can be used for other species of interest.

In both subjects, colour discrimination thresholds (Table 1) were similar to other trichromatic Platyrrhini [20] and were higher than human thresholds [26]. It is still unclear whether this threshold difference compared to humans is due to a limitation of the subject’s behavior or a consequence of the tests used based on human colour space. In our experiment, the female *A. seniculus* also showed a slightly higher threshold than the male *A. caraya* (Fig. 2A,B). However, this could be a reflex of her poorer performance during the procedure, as she was constantly aware of surrounding movements and noises, which led to mistakes even when aiming for the correct stimulus in every session. Nevertheless, the discrimination ellipses of both subjects were round and different from the elongated ellipses displayed by the dichromat male capuchin ellipse shown in the Fig. 2C.

The behavioural evaluation in this study shows evidence of a uniform trichromacy in *A. caraya* and *A. seniculus*, with thresholds similar to those reported by Goulart and collaborators [20] in trichromatic females of capuchin monkeys (Fig. 2C), in agreement with previous results from Araújo Jr. and collaborators [15] with *A. caraya*. Therefore, even with the unique evolutionary changes in this genus that led to uniform trichromacy and a different photoreceptor distribution in the retina [10], those changes resulted in a colour vision phenotype similar to that found in Catarrhini.

Pseudoisochromatic tests minimize perceptual cues that a subject can use, such as brightness and border contrast, by using a mosaic of components with random spatial and luminance noise, in which the target differs from the background only by chromatic cues [27]. Even if potentially indistinguishable for a dichromatic observer, two adjacent patches of different colours could be perceived differently due to the Helmholtz-Kourash effect or border artifact [28]. Along with those principles, our behavioural experiment compares a reference chromaticity to several surrounding chromaticities in CIE 1976 chromaticity diagram, obtaining a threshold value of discrimination distance (vector length) between each tested chromaticity and the background. This allows us to generate a MacAdam ellipse [29], that represents the confusion region for the given background chromaticity, providing more information compared to tests with fewer confusion pairs. This robustness is valuable for the study of colour discrimination in Platyrrhini due to the presence of high degree of polymorphisms and hybrid genes in populations of several species, which could be easily missed with a qualitative test or using tests with fewer chromatic axes, as in most electrophysiological and simpler behavioural tests for colour vision evaluation [30].

However, the behavioral evaluation has some limitations as it takes a long time to train the subjects. The necessity of an expensive and adapted equipment also limits the mobility and restricts the experiment to one housing facility. *Alouatta* genus availability is also restricted and a large study group is hard to find. These limitations are reflected in our study that included a low sample size of one male and one female from this genus, which restricted our capability of evaluating populational effects.

Howler monkeys are distinct from other Platyrrhini and even within the *Atelidae* family, in terms of colour vision. In the *Atelidae* family, the *Alouatta* genus was the first to diverge (16 Ma), followed by the genera *Ateles* (11.5 Ma), and by *Brachyteles* and *Lagothrix* (9.5 Ma) [31]. The genus *Alouatta* had several speciation episodes over their evolution, until recent times, making modern species very different from their ancestor and closely related species [32]. Rosenberger and collaborators [33] stated that the cranio-dental features of the genus has been regarded as a complex adaptation to the increased folivore diet and the use of howling for communication. Youlatos, Couette and Halenar [6] also highlighted behaviours associated with energy saving as triggers for the adaptations of the *Alouatta* genus [6].

Howler monkeys’ diet is classified as folivorous-frugivorous, including flowers, lichens, stems and tree barks. This is a low energy diet that may have led to behavioural adaptations to save energy [34, 35]. Several howler monkey species show a preference for young and more reddish leaves, and thus, trichromacy would benefit the discrimination of red from green at a distance [1, 36]. Melin and colaborators [14] also showed a positive correlation between howler monkeys trichromatic and anomalous trichromatic hypothesized colour vision and an improved discrimination of younger leaves. This could be a theoric evidence of the adaptive importance of the trichromatic colour vision for the preference of howler monkeys for younger and reddish leaves.. Our work provides useful insights for studies about colorimetric information on food consumption by these subjects. Such information can be compared to quantitative color discrimination data from *Alouatta* genus and help determine the extent to which chromatic cues are used on food preference. Other highlighted visual differences between *Alouatta* and other Platyrrhini pointed to a compaction of cones in the central area of the retina, resulting in the highest cone concentration found in primate retinas [8–12]. However, this compaction did not result in an improvement in colour discrimination compared to other Platyrrhini trichromats (Fig. 2D), and the extent of its impact in other visual functions still needs to be evaluated.

## Conclusion

We successfully performed a behavioural colour discrimination test on a male *A. caraya* and a female *A. seniculus*, and fit their colour discrimination ellipses for three different chromaticity backgrounds in the CIE 1976 chromaticity diagram. Our behavioural data showed evidence of uniform trichromacy among males and females in the *Alouatta* genus, and we found no evidence that the higher photoreceptor density in the central retina described in previous studies resulted in quantifiable difference in colour vision discrimination compared to other primates. Our genetics findings also support the presence of both *mws* and *lws* cone opsin genes in a male *A. caraya* and a female *A. seniculus.*

## Methods

### Ethics statement

All protocols and animal care procedures were performed according to local and international ethical guidelines (NIH Publications No. 8023, revised 1978) and were approved by the Brazilian environment agency (SISBIO 32763-3) and by the Animal Research Ethics Committee (CEPAE) of the National Primate Center, in Pará, Brazil (CENP-PA) (CEPAN n°33/2011). The monkeys were not harmed during the study.

### Subjects

**Fig. 3:**
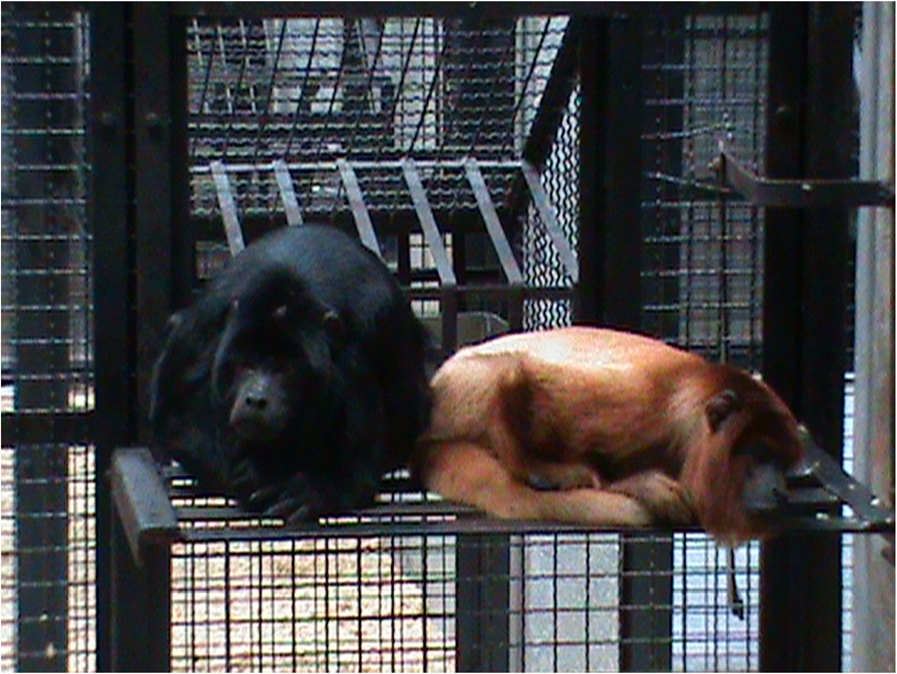
Subjects and housing conditions. A male *A. caraya* (on the left) and a female *A. seniculus* (on the right), housed at the National Primate Center (CENP), Ananindeua, Pará, Brazil

Six *Alouatta* spp. individuals, housed at the Brazilian National Primate Center (CENP-PA) were trained for the behavioural tests: four adult males (three *A. caraya* and one *A. belzebul*) and two females, one adult (*A. seniculus*) and one juvenile (*A. belzebul*) (Fig. 3). Subjects had no previous history of reinforcement and some were resistant, as they were rescued from wildlife, from distress conditions, habitat loss or hunter’s captivity, and were not used to human handling. Animals were kept in enclosures (2.5 m wide × 4 m deep × 2.3 m high) connected through a window to twin enclosures. The enclosures were exposed to normal fluctuations in photoperiod, with a cycle of approximately 12 h daylight and 12 h dark. Subjects were fed twice a day with primate ration and fruits, leaves daily, juice or milk twice a week, and water was provided ad libitum. The subjects were not food deprived prior to the experiment, having it removed only during the test sessions.

### Blood samples collection

Blood samples were collected by specialized staff from the CENP-PA. The procedure followed local routine with anesthetic association of tiletamine and zolazepam (10 mg/kg). Approximately 6 ml of blood was collected from the femoral vein with a 22G hypodermic needle adapted to a BD Vacutainer® tube with 3.2% buffered sodium citrate solution. After blood collection, animals were returned to an empty enclosure until full recovery.

### Genetic analysis

#### DNA extraction, PCR, and sequencing

Genetic analyzes were performed to investigate the *lws*, *mws*, and *sws1* opsin genes of two *Alouatta* species, *A. seniculus* and *A. caraya*. Genomic DNA was extracted from the blood samples using standard protocols (Puregene DNA®, Gentra System). Polymerase chain reactions (PCRs) were carried out to amplify exons 3 and 5 of the *lws* and *mws* opsin genes, and the exon 1 of the *sws1* opsin gene, to identify the specific amino acid residues responsible for the opsins spectral tuning. The primers used to amplify the *lws/mws* and the *sws1* exons were described elsewhere [18, 37]; (Supplementary Table S1). PCRs were performed using High Fidelity Platinum Taq Polymerase, and PCR conditions are described in the Supplementary Table S2. The PCR products were visualized by agarose gel electrophoresis (2%), and purified using the Illustra™ GFX PCR DNA and Gel Band purification kit (GE Healthcare), according to the manufacturer protocol. The purified samples were directly sequenced in both directions, using BigDye Terminator v3.1 Cycle Sequencing Kit (Applied Biosystem™) and a 3500xL sequencer (Applied Biosystem™), and sequences were analyzed with BioEdit [38].

#### Molecular cloning and inter-exon PCR

Based on the variations observed from the sequencing results, we confirmed that both opsin genes *mws* and *lws* were amplified together by the primer pairs used. In order to sequence the two genes separately, PCR products obtained from amplification of exon 5 from the two samples (*A. seniculus* and *A. caraya*), were cloned into plasmid vectors using TA cloning® kits (Invitrogen). Multiple clones of each sample were isolated, purified via spin columns (Qiagen), and sequenced using M13 vector primers. Based on sequences analyses from 10 individual clones of each sample, we were able to differentiate exon 5 of the *mws* and the *lws* genes, and to design specific primers for each gene. In an inter-exon PCR, we combined a common forward primer for both genes located in exon 3, with reverse primers specific for the *mws* and for the *lws* genes, located at exon 5 (Supplementary Table S1). The PCRs were performed using the enzyme TaKaRa LA Taq® (Takara Bio USA), and PCR parameters were based on Davidoff, Neitz & Neitz [39] (Supplementary Table S2). The PCR products over 2 kb were visualized using a 2% agarose gel, purified, and sequenced in both directions as described above. Sequences were analyzed with BioEdit 7.25 [38], and exons 3 and 5 of each gene were identified.

#### Estimates of the absorption peaks of the LWS, MWS, and SWS1 opsins

The opsin sequences were aligned with the corresponding sequences of other primates and spectral tuning sites were identified to estimate the opsins λ_max_. For the SWS1 opsin, amino acids were numbered based on the bovine rhodopsin (RH1) (GenBank accession number NM_001014890), and residues 46, 49, 52 81, 86, 90, 93, 114, 116, and 118, were considered for spectral tuning estimates [40]. For the LWS and MWS opsins, sequences were aligned with human LWS opsin to identify the spectral tuning sites 180, 277, and 285, and estimate the opsins λ_max_ [22, 41–43].

### Behavioural tests

#### Apparatus

The behavioural colour discrimination evaluation was performed in a computerized system consisting of a video monitor (Diamond Pro 2070SB, Mitsubishi, Cypress, CA, USA, spatial resolution: 2048 × 1536 at 86 Hz -, frame rate: 160 Hz, colour resolution: 14 bits) in which the stimuli were displayed on a video monitor adapted to receive touch inputs. A software programmed the experimental sessions and recorded the monkey’s responses. The setup (ViSaGe system® - Cambridge Research Systems, Rochester, UK, and CRT monitor), previously used and described in detail by Goulart and collaborators [20], was adapted for *Alouatta* subjects, with a larger food dispenser and tray. Gamma correction was performed to calibrate the luminance of the monitor guns using the software VSG Desktop (CRS) and a *ColorCAL MKII Colorimeter* (Cambridge Research Systems, Rochester, UK). The monitor was placed at a distance of 20 cm from the subjects’ eyes, allowing them to reach the target only with the fingers. For this experiment, the equipment was mounted on a mobile rack, allowing in situ experiments, attached to a 60 × 60 × 60 cm experimental chamber.

#### Stimulus

The stimulus was an adaptation by Goulart and collaborators [24], of the Cambridge Colour Test (CCT) (Cambridge Research Systems, Rochester, UK), in which the Landolt-C target is replaced by a composite image resembling a 5 cm square patch that can appear in one of four different positions (top left, top right, bottom left, bottom right). Both, target and background, were composed of a matrix of circles of random diameter, ranging from 5 to 9 mm, and random luminance, ranging from 7 and 15 cd/m^2^. The CIE 1976 chromaticity diagram coordinates for the background chromaticity were u’ = 0.1977, v’ = 0.4689 for the first ellipse (E1), u’ = 0.1925, v’ = 0.5092, for the second ellipse (E2) and u’ = 0.2044, v’ = 0.4160 for the third ellipse (E3). In each trial, target chromaticity changed dynamically, with the target varying within one of the 20 predefined chromatic vectors. We used a staircase procedure to estimate colour discrimination threshold for each vector. The excursion along each vector varied between 1100 × 10^− 4^ and 20 × 10^− 4^ u’v’ units, getting one step closer to the background chromaticity for every correct response and one step away for wrong responses. Discrimination thresholds, expressed as threshold vector length in u’v’ units in 1976 CIE chromaticity diagram, were obtained along 20 equally spaced chromatic vectors surrounding the reference chromaticity, by an average of the last seven of 11 reversals. We considered reversals wrong responses followed by correct ones, and correct responses followed by wrong ones. MATLAB routines were used to interpolate these thresholds resulting in an ellipse around the background chromaticity.

#### Procedures

##### General procedures

**Fig. 4:**
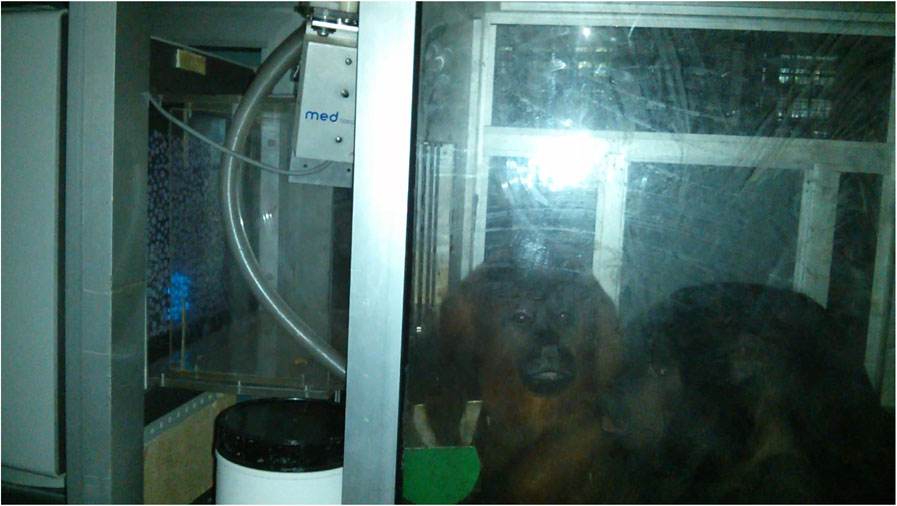
Subjects during training procedure in the apparatus. Both subjects a male *A. caraya* (black pelage) and a female *A. seniculus* (red pelage) inside the experimental chamber during training session. The monitor on the left displays a stimulus similar to the test stimulus with the target on the bottom surrounded by an isochromatic field composed of the same pattern of circles

All subjects were trained 3 days a week via positive reinforcement through a shaping procedure (Fig. 4). Session duration was up to an hour. They were first adapted to the presence of the researcher, to the type of reinforcement – banana flavored pellets (Bioserv®, Flemington USA), grapes or nuts – and to the test apparatus. Following this phase, subjects were trained through several steps with increasing complexity from touching the monitor to a stage where they consistently discriminated a colour patch from the background. Each trial began with the presentation of the stimulus and ended with its disappearance, after a touch response hit any point on the screen. Touch responses on the target area were immediately followed by reward presentation. A 5 s intertrial interval (ITI) was programmed before the following stimulus presentation. Touch responses on any other part of the screen were followed by the ITI alone.

##### Training phase

During training, target chromaticities were highly saturated distant from any confusion lines predicted for dichromats in the CIE 1976 chromaticity diagram, deliberately facilitating target detection for training purposes. At the beginning of the training, the target-background distance was kept constant at the maximum (1100 × 10^− 4^ u’v’ units). When subjects learned to consistently touch over the target, we introduced the dynamic staircase variation of the target-background distance, with the minimum distance of target to background chromaticity defined as 200 × 10^− 4^ u’v’ units. The minimum chromatic target-background distance gradually changed after each successful training day from the easy training parameters (minimum distance of 200 × 10^− 4^ u’v’ units) to the test parameters (minimum distance of 20 × 10^− 4^ u’v’ units). Once subjects reached three consecutive days of consistent discrimination at the minimum distance, the training was considered successful, and they received a pretest session that simulated the test conditions. In the test simulation, subject was presented with three novel vectors (outside hypothesized confusion lines), and the minimum distance was set at 20 × 10^− 4^ u’v’ units, which would eventually lead to failure to distinguish target from background, at the threshold region.

##### Testing phase

Subjects were tested along 20 chromatic vectors for each ellipse background chromaticity, defined around the same background coordinates used by the Cambridge Colour Test. A test session presented five out of 20 vectors, and the whole test consisted of four sessions for each background chromaticity. The staircase was automatically generated, with upper and lower limits respectively at 1100 × 10^− 4^ u’v’ units and 20 × 10^− 4^ u’v’ units. When the subjects’ performance reached the criterion of 11 reversal trials, a discrimination threshold for that vector was derived from the average distances between the background chromaticity and the target chromaticities at which the last seven reversals occurred. Chromatic discrimination thresholds coordinates were fitted to an ellipse model and the parameters of the ellipses (major axis length, axis ratio, and angle) guided the behaviourally based phenotypic classification.

## Supplementary Information

**Additional file 1.**

## Data Availability

The datasets used and/or analysed during the current study are available from the corresponding author on reasonable request.
