# Supplementary material for: Uniform trichromacy in Alouatta caraya and Alouatta seniculus: behavioural and genetic colour vision evaluation
Source: Front Zool. 2021 Jul 8;18:36. doi: 10.1186/s12983-021-00421-0 (PMC8268213; doi:10.1186/s12983-021-00421-0)
Supplement: Supplementary file 1 — Additional file 1. [file 12983_2021_421_MOESM1_ESM.docx]

**SUPPLEMENTARY TABLE S1**

| SWS1 Exon 1 Primers | |
| --- | --- |
| SWS1X1_MNK_Fw2 | AAGAGGACTCAGAGGAGGGTGTG |
| SWS1X1_MNK_Rv1 | CTAACCCCTTTTTCCCCTGC |

| M/LWS Exon 3 Primers | |
| --- | --- |
| Mnkx3 Fw | GGATCACGGGTCTCTGGTC |
| Mnkx3 Rv | CTGCTCCAACCAAAGATGG |

| M/LWS Exon 5 Primers | |
| --- | --- |
| Cebus5x5 | GTGGCAAAGCAGCAGAAAG |
| Cebus3x5 | CTGCCGGTTCATAAAGACATAG |

| Long-Range PCR Primers | |
| --- | --- |
| Acar_X3-5’ | GGATCACGGGTCTCTGGT |
| Acar_LWS_X5-3’ | CCCCAGCAGATGCAGTAAGCC |
| Acar_MWS_X5-3’ | GCAGACGCAGAACGCTAT |

**SUPPLEMENTARY TABLE S2**

| **Protocol SWS1 Exon 1** | | |
| --- | --- | --- |
| Degrees | Time | Cycles |
| 94 | 1 min | 1x |
| 94 | 15 sec | 37 |
| 60 | 30 sec |  |
| 72 | 30 sec |  |
| 72 | 7 min | 1x |
| 4 | hold |  |

| **Protocol M/LWS Exon3** | | |
| --- | --- | --- |
| Degrees | Time | Cycles |
| 94 | 5 min | 1x |
| 94 | 15 sec | 37 |
| 59 | 30 sec |  |
| 72 | 45 sec |  |
| 72 | 20 min | 1x |
| 4 | hold |  |

| **Protocol M/LWS Exon5** | | |
| --- | --- | --- |
| Degrees | Time | Cycles |
| 94 | 5 min | 1x |
| 94 | 15 sec | 37 |
| 63 | 30 sec |  |
| 72 | 45 sec |  |
| 72 | 20 min | 1x |
| 4 | hold |  |

| **Protocol M/LWS Exon3-Exon5** | | |
| --- | --- | --- |
| Degrees | Time | Cycles |
| 94 | 3 min | 1x |
| 94 | 30 sec | 30 |
| 59 | 30 sec |  |
| 68 | 6 min |  |
| 68 | 20 min | 1x |
| 4 | hold |  |

The Invitrogen Platinum Taq kit was used for the single exons samples. DNA segments larger than 2 kb were amplified with Takara LA Taq kit (Clonetech).
